# Mining and analysis of adverse events for trastuzumab deruxtecan based on the FAERS database

**DOI:** 10.3389/fonc.2026.1778579

**Published:** 2026-05-29

**Authors:** Anqing Zhu, Rilei Jiang, Chong Zhou, Jian Dong, Cheng Huang, Minjie Zhu, Jianlong Gu, Jun Xu, Jianzhong Ji, Haohao Zhu, Yiting Cao

**Affiliations:** 1 Jiangsu Medical College, Yancheng, Jiangsu, China; 2 School of Chinese Medicine, Shanghai University of Traditional Chinese Medicine, Shanghai, China; 3 Jiangsu Key Laboratory of New Drug Research and Clinical Pharmacy, Xuzhou Medical University, Xuzhou, Jiangsu, China; 4 Department of Radiotherapy, Xuzhou Central Hospital, Xuzhou, Jiangsu, China; 5 Sheyang Hospital of Traditional Chinese Medicine, Yancheng, Jiangsu, China; 6 Affiliated Mental Health Center of Jiangnan University, Wuxi Central Rehabilitation Hospital, Wuxi, Jiangsu, China; 7 Department of Molecular Biology and Genetics, Weill Institute for Cell and Molecular Biology, Cornell University, Ithaca, NY, United States

**Keywords:** adverse event, drug safety monitoring, FAERS database, HER2-positive cancer treatment, trastuzumab deruxtecan

## Abstract

**Objective:**

This study aims to mine and analyze adverse event (AE) signals for trastuzumab deruxtecan (T-DXd) using the FAERS database, particularly focusing on AEs that are not fully described in clinical trials and early market surveillance.

**Methods:**

The study selected data from the first quarter of 2020 to the third quarter of 2023, searching with T-DXd as the primary suspected drug. Four advanced signal mining and analysis methods were employed: Reporting Odds Ratio (ROR), Proportional Reporting Ratio (PRR), Bayesian Confidence Propagation Neural Network (BCPNN), and Empirical Bayesian Geometric Mean (EBGM), to comprehensively assess the AE signals in the FAERS database.

**Results:**

The study collected 6,703,410 AE reports, of which 4,055 reports involved T-DXd. Through comprehensive analysis, 118 PT signals involving 26 SOCs were identified. The study revealed various newly discovered AEs associated with T-DXd treatment, including immune dysregulation events like Gastroenteritis listeria and Coronavirus pneumonia, hepatobiliary disorders like Pseudocirrhosis and Hepatic failure, and hematological issues like Neutropenia and Thrombocytopenia. Additionally, new safety signals for cardiovascular and ocular problems were found.

**Conclusion:**

This study confirmed known AEs and identified a range of new AEs, aiding clinicians in making more informed decisions when using T-DXd.

## 1 Introduction

Breast cancer (BC) is the most commonly diagnosed malignancy and the leading cause of cancer-related death among women globally, with an estimated 2.3 million new cases and 685,000 deaths reported in 2020 (1). Treatment decisions for BC are primarily guided by molecular subtyping based on the expression of estrogen receptor (ER), progesterone receptor (PR), and human epidermal growth factor receptor 2 (HER2) (2). Common Approximately 15–20% of BC cases are classified as HER2-positive, a subtype historically associated with aggressive tumor biology and poor prognosis before the advent of targeted therapies (3). The development of anti-HER2 agents—beginning with trastuzumab in 1998—has revolutionized the treatment landscape for HER2-positive BC, significantly improving survival outcomes in both early and metastatic settings (4). These drugs, after continuous treatment regimen optimization, have been widely used in adjuvant therapy, first-line treatment, or multi-line treatment. Since the introduction of drugs like Trastuzumab (Herceptin) and Lapatinib (Tykerb), the treatment landscape for patients with HER2-positive BC has changed significantly, with anti-HER-2 treatments notably delaying the progression of this subtype of tumors (5).

ADCs represent a novel class of targeted therapeutics that combine the antigen specificity of monoclonal antibodies with the potent cytotoxicity of chemotherapeutic agents, earning them the moniker “biological missiles”. Unlike conventional chemotherapy, ADCs deliver cytotoxic payloads directly to HER2-expressing tumor cells, minimizing off-target toxicity to normal tissues. However, the structural complexity of ADCs—encompassing the monoclonal antibody backbone, cleavable/non-cleavable linkers, and cytotoxic payloads—can lead to unique and potentially severe adverse events (AEs) distinct from those of traditional chemotherapy or monoclonal antibodies (6). Therefore, for the clinical use of ADC drugs, it is essential to focus on, monitor, and manage their potential adverse reactions.

T-DXd is a next-generation ADC that was approved in December 2019 (7). It consists of trastuzumab linked to a topoisomerase I inhibitor via a cleavable tetrapeptide linker. Compared to its predecessor, T-DM1, T-DXd has a higher drug-to-antibody ratio, which allows for more effective tumor cell killing. Additionally, the released DXd can penetrate surrounding cells with low HER2 expression, producing a “bystander effect.” Therefore, T-DXd is not only approved for the treatment of HER2-positive breast cancer but is also used in the clinical treatment of other solid tumors, such as gastric cancer, colorectal cancer, and non-small cell lung cancer (8).With the widespread clinical use of T-DXd, its safety and tolerability issues have become more apparent. Some patients have experienced adverse reactions, including pulmonary complications, cardiac issues, and other potential side effects, after treatment (9). These observations indicate a need for more in-depth research and monitoring of the drug’s safety.

The FDA Adverse Event Reporting System (FAERS) is designed to collect and monitor AE reports during the use of pharmaceuticals (10–15). This study aims to conduct a comprehensive analysis of AE reports for T-DXd based on the FAERS database, to provide more exhaustive and reliable safety data for this drug.

## 2 Materials and methods

### 2.1 Data source

The data for this study were sourced from the FAERS database, which is updated quarterly and includes suspected AEs from the United States and other countries. These reports encompass various information related to AEs, such as indications for drug use, patient demographics, types of AEs, occurrence time, duration, and outcomes. The study selected data from the first quarter of 2020 to the third quarter of 2023.

### 2.2 Data processing

This study utilized the SAS software package to extract, clean, analyze, and mine data from the FAERS database. Reports where TRASTUZUMAB DERUXTECAN was the primary suspected drug were retrieved. Extracted data included patient demographics, drug utilization, adverse reaction terms, and patient outcomes. For standardization and classification of AEs, the study adhered to the Preferred Term (PT) and System Organ Class (SOC) as per the Medical Dictionary for Regulatory Activities (MedDRA) (16).

### 2.3 Data analysis

The study employed disproportionality analysis methods for signal mining, including frequency and Bayesian methods. Frequency methods encompassed the Reporting Odds Ratio (ROR) and Proportional Reporting Ratio (PRR); Bayesian methods included the Bayesian Confidence Propagation Neural Network (BCPNN) and Empirical Bayesian Geometric Mean (EBGM) (17–20). Calculations for both methods were based on 2x2 contingency table data (**Table 1**). The study defined the threshold criteria for positive signals (**Table 2**). To reduce false-positive signals, an association between the drug and the AE was deemed related only if it met the above criteria and the frequency of the AE occurrence was three or more cases.

**Table 1:** Four grid table.

Exposure	Target AEs	Non-target AEs	Total
T-DXd	a	b	a+b
Non- T-DXd	c	d	c+d
Total	a+c	b+d	N=a+b+c+d

**Table 2:** ROR, PRR, BCPNN, and EBGM methods, formulas, and thresholds.

Method	Formula	Threshold
ROR	$\text{ROR}=\frac{\left(a/c\right)}{\left(b/d\right)}=\frac{ad}{bc}$ $\text{SE}\left(\text{lnROR}\right)=\sqrt{\left(\frac{1}{\text{a}}+\frac{1}{\text{b}}+\frac{1}{\text{c}}+\frac{1}{\text{d}}\right)\;}$ $95\text{\%CI}={\text{e}}^{\text{ln}\left(\text{ROR}\right)\pm 1.96\sqrt{\;\left(\frac{1}{\text{a}}+\frac{1}{\text{b}}+\frac{1}{\text{c}}+\frac{1}{\text{d}}\right)\;}}$	a ≥ 3 and 95% CI (lower limit) > 1
PRR	$\text{PRR}=\frac{a/\left(a+b\right)}{c/\left(c+d\right)}$SE(lnPRR)= $\sqrt{\;\frac{1}{a}-\frac{1}{a+b}+\frac{1}{c}-\frac{1}{\text{c}+\text{d}}\;\;}$ 95%CI = $e^{\text{ln}\left(\text{PRR}\right)\pm 1.96\sqrt{\;\frac{1}{a}-\frac{1}{a+b}+\frac{1}{c}-\frac{1}{\text{c}+\text{d}}\;\;}}$	a ≥ 3 and 95% CI (lower limit) > 1
BCPNN	IC= $log_{2}\frac{p\left(x,y\right)}{p\left(x\right)p\left(y\right)}=log_{2}\frac{a\left(a+b+c+d\right)}{\left(a+b\right)\left(a+c\right)}$E(IC)= $log_{2}\frac{\left(a+\gamma 11\right)\left(a+b+c+d+\alpha \right)\left(a+b+c+d+\beta \right)}{(a+b+c+d+\gamma )\left(a+b+\alpha 1\right)\left(a+c+\beta 1\right)}$V(IC)= $\frac{1}{{\left(ln2\right)}^{2}}\left\{\left[\frac{\left(a+b+c+d\right)-a+\gamma -\gamma 11}{\left(a+\gamma 11\right)\left(1+a+b+c+d+\gamma \right)}\right]+\left[\frac{\left(a+b+c+d\right)-\left(a+b\right)+\alpha -\alpha 1}{\left(a+b+\alpha 1\right)\left(1+a+b+c+d+\alpha \right)}\right]+\left[\frac{\left(a+b+c+d\right)-\left(a+c\right)+\beta -\beta 1}{\left(a+c+\beta 1\right)\left(1+a+b+c+d+\beta \right)}\right]\right\}$ $\gamma =\gamma 11\frac{\left(a+b+c+d+\alpha \right)\left(a+b+c+d+\beta \right)}{\left(a+b+\alpha 1\right)\left(a+c+\beta 1\right)}$*IC-2SD=E(IC)-2* $\sqrt{V\left(IC\right)}$	IC025>0
EBGM	$\text{EBGM}=\frac{a\left(a+b+c+d\right)}{\left(a+c\right)\left(a+b\right)}$ $95\text{\%CI}={\text{e}}^{\text{ln}\left(\text{EBGM}\right)\pm 1.96\sqrt{\;\left(\frac{1}{\text{a}}+\frac{1}{\text{b}}+\frac{1}{\text{c}}+\frac{1}{\text{d}}\right)\;}}$	EBGM05 > 2

### 2.4 Signal classification and validation

To objectively distinguish novel unlabeled AEs from known labeled AEs, we used the FDA-approved prescribing information (PI) for T-DXd as the sole reference standard for labeled AEs. We systematically extracted all AE-related content from the following sections of the PI: Boxed Warnings, Warnings and Precautions, Adverse Reactions (including both clinical trial and post-marketing reports), Drug Interactions (if relevant to AE development). Two independent investigators performed the label mapping and AE classification separately. Discrepancies were resolved through consensus discussion with a third senior investigator. All final classification decisions were reviewed and approved by the entire study team.

## 3 Results

From the first quarter of 2020 to the third quarter of 2023, the FAERS database collected a total of 6,703,410 AE reports, with 4,055 involving T-DXd. Through integrated analysis using ROR, PRR, BCPNN, and EBGM methods, 118 PT signals involving 26 SOCs were identified. This large volume of data provides a foundation for a comprehensive assessment of T-DXd’s safety.

### 3.1 Basic information on AE reports

The data show that among the reported patients in **Table 3**, females (66.39%) were more common than males (11.89%). Despite a significant proportion of missing age data (61.09%), among reports with provided age information, the highest proportion was patients aged 45 to 65 years (18.25%). Since 2020, there has been a yearly increase in the number of reports. Regarding the identity of the reporters, healthcare professionals were the primary source, with physicians accounting for 58.91% of the reports, followed by pharmacists (26.93%) and consumers (14.08%). In terms of national distribution, the majority of reports were from the United States (53.42%), followed by Japan, Canada, France, and Italy. Regarding severe AEs, 23.33% of the reports involved death. Among cases where the event occurrence time was clearly reported, the peak period for AEs was within the first 30 days after medication, accounting for 13.51%.

**Table 3:** Basic information on AEs related to T-DXd.

Factors	ftNumber of events (%)
Gender	
Female	2692(66.39)
Male	482(11.89)
Unknown	881(21.73)
Age	
<18	1( 0.02)
18-45	198( 4.88)
45-65	740(18.25)
65-75	422(10.41)
≥75	217( 5.35)
Unknown	2477(61.09)
Reporter	
Consumer	571(14.08)
Physician	2389(58.91)
Pharmacist	1092(26.93)
Other Health Professionals	2( 0.05)
Lawyer	1( 0.02)
Reported Countries	
United States	2166(53.42)
Japan	589(14.53)
Canada	415(10.23)
France	258( 6.36)
Italy	88( 2.17)
Report Year	
2020	373( 9.20)
2021	631(15.56)
2022	1323(32.63)
2023	1728(42.61)
Serious Outcomes	
Death	946(23.33)
Disability	30( 0.74)
Hospitalization - Initial or Prolonged	1045(25.77)
Life-Threatening	169( 4.17)
AE occurrence time - medication date (days)	
0-30	548(13.51)
31-60	113( 2.79)
61-90	78( 1.92)
91-120	55( 1.36)
121-150	48( 1.18)
151-180	31( 0.76)
181-360	101( 2.49)
>360	40( 0.99)

### 3.2 AE signal mining results

At the SOC level, AEs related to T-DXd involved 26 SOCs, detailed in **Table 4**. The most common SOCs included General disorders and administration site conditions, Gastrointestinal disorders, and Respiratory, thoracic and mediastinal disorders, consistent with the information in the drug’s label. Injury, poisoning and procedural complications, Infections and infestations, Hepatobiliary disorders, Cardiac disorders, and Psychiatric disorders, though not explicitly mentioned in the label, showed higher frequencies of occurrence, with Hepatobiliary disorders in particular warranting further attention due to their signal strength.

**Table 4:** The signal strength of AEs of T-DXd at the SOC level.

System organ class	SOC code	Case reports	ROR (95% CI)	PRR (95% CI)	χ2	IC (IC025)	EBGM (EBGM05)
General disorders and administration site conditions	10018065	2171	1.27 (1.21-1.34)	1.21 (1.17-1.26)	100.06	0.28 (0.21)	1.21 (1.16)
Gastrointestinal disorders	10017947	1508	2.10 (1.98-2.21)	1.93 (1.84-2.02)	733.13	0.95 (0.87)	1.93 (1.83)
Respiratory, thoracic and mediastinal disorders	10038738	1274	3.11 (2.93-3.30)	2.84 (2.70-2.99)	1590.34	1.50 (1.42)	2.84 (2.68)
Injury, poisoning and procedural complications	10022117	960	0.76 (0.71-0.82)	0.79 (0.74-0.84)	63.46	-0.35 (-0.44)	0.79 (0.74)
Investigations	10022891	738	1.27 (1.18-1.37)	1.25 (1.17-1.34)	39.08	0.32 (0.21)	1.25 (1.16)
Blood and lymphatic system disorders	10005329	570	3.54 (3.25-3.85)	3.40 (3.13-3.68)	977.54	1.76 (1.63)	3.39 (3.12)
Infections and infestations	10021881	511	0.90 (0.83-0.99)	0.91 (0.84-0.99)	4.84	-0.14 (-0.27)	0.91 (0.83)
Metabolism and nutrition disorders	10027433	398	2.14 (1.93-2.36)	2.09 (1.90-2.30)	231.29	1.06 (0.91)	2.09 (1.89)
Nervous system disorders	10029205	363	0.48 (0.43-0.53)	0.50 (0.45-0.55)	196.29	-1.00 (-1.15)	0.50 (0.45)
Skin and subcutaneous tissue disorders	10040785	297	0.53 (0.47-0.59)	0.54 (0.49-0.61)	120.73	-0.88 (-1.05)	0.54 (0.48)
Neoplasms benign, malignant and unspecified (incl cysts and polyps)	10029104	286	0.62 (0.55-0.70)	0.63 (0.57-0.71)	62.89	-0.65 (-0.83)	0.63 (0.56)
Hepatobiliary disorders	10019805	194	2.43 (2.11-2.80)	2.40 (2.09-2.76)	159.56	1.25 (1.04)	2.40 (2.08)
Cardiac disorders	10007541	118	0.59 (0.50-0.71)	0.60 (0.50-0.72)	32.43	-0.74 (-1.00)	0.60 (0.50)
Musculoskeletal and connective tissue disorders	10028395	116	0.22 (0.18-0.26)	0.23 (0.19-0.27)	323.13	-2.13 (-2.40)	0.23 (0.19)
Surgical and medical procedures	10042613	106	0.73 (0.61-0.89)	0.74 (0.61-0.89)	10.05	-0.44 (-0.72)	0.74 (0.61)
Psychiatric disorders	10037175	97	0.17 (0.14-0.20)	0.17 (0.14-0.21)	403.66	-2.51 (-2.81)	0.17 (0.14)
Renal and urinary disorders	10038359	90	0.47 (0.38-0.57)	0.47 (0.38-0.58)	54.59	-1.08 (-1.38)	0.47 (0.38)
Eye disorders	10015919	87	0.45 (0.36-0.55)	0.45 (0.37-0.56)	59.00	-1.14 (-1.45)	0.45 (0.37)
Vascular disorders	10047065	79	0.42 (0.34-0.52)	0.42 (0.34-0.53)	62.92	-1.23 (-1.55)	0.42 (0.34)
Immune system disorders	10021428	24	0.21 (0.14-0.31)	0.21 (0.14-0.32)	70.73	-2.19 (-2.76)	0.21 (0.14)
Social circumstances	10041244	24	0.50 (0.33-0.74)	0.50 (0.33-0.74)	12.12	-0.97 (-1.55)	0.50 (0.33)
Ear and labyrinth disorders	10013993	17	0.41 (0.26-0.67)	0.42 (0.26-0.67)	14.02	-1.22 (-1.90)	0.42 (0.26)
Product issues	10077536	14	0.07 (0.04-0.13)	0.08 (0.05-0.13)	160.01	-3.62 (-4.37)	0.08 (0.05)
Endocrine disorders	10014698	11	0.43 (0.24-0.77)	0.43 (0.24-0.77)	8.54	-1.16 (-1.99)	0.43 (0.24)
Reproductive system and breast disorders	10038604	9	0.15 (0.08-0.28)	0.15 (0.08-0.28)	45.27	-2.65 (-3.56)	0.15 (0.08)
Congenital, familial and genetic disorders	10010331	5	0.20 (0.08-0.48)	0.20 (0.08-0.48)	15.84	-2.10 (-3.28)	0.20 (0.08)

At the PT level, results were ranked according to the number of reports and signal strength, with the top 30 PTs listed in **Tables 5** and **6**. In **Table 5**, the top 10 PTs with the most reports mostly consisted of AEs already recorded in the label. In addition to known AEs, the study also found high frequencies of Neutropenia, Thrombocytopenia, Ascites, Pleural effusion, Dehydration, Pulmonary embolism, and Respiratory failure. In **Table 6**, Gastroenteritis listeria and Coronavirus pneumonia were among the top three and not recorded in the drug label, potentially representing new AEs. Conditions like Focal nodular hyperplasia, Pseudocirrhosis, Breast hemorrhage, Lymphangiosis carcinomatosa, Eye hematoma, Radiation necrosis, Metastases to bone marrow, Malignant ascites, Metastases to skin, although less frequently reported, showed higher signal strengths, suggesting these AEs warrant further in-depth research and analysis.

**Table 5:** Top 30 PTs ranked by report numbers.

SOC	PTs	Case reports	ROR (95% CI)	PRR (95% CI)	χ2	IC (IC025)	EBGM (EBGM05)
Gastrointestinal disorders	Nausea	554	5.15 (4.72-5.61)	4.92 (4.53-5.33)	1743.57	2.28 (2.16)	4.91 (4.50)
Respiratory, thoracic and mediastinal disorders	Interstitial lung disease	430	62.12 (56.31-68.53)	59.51 (54.16-65.38)	23919.13	5.67 (5.52)	57.53 (52.15)
Injury, poisoning and procedural complications	Off label use	423	2.41 (2.19-2.66)	2.35 (2.14-2.58)	333.68	1.23 (1.08)	2.35 (2.13)
General disorders and administration site conditions	Death	418	3.00 (2.72-3.30)	2.91 (2.65-3.20)	531.72	1.53 (1.39)	2.91 (2.64)
General disorders and administration site conditions	Disease progression	410	21.22 (19.21-23.43)	20.39 (18.54-22.43)	7487.07	4.27 (4.12)	20.16 (18.26)
General disorders and administration site conditions	Fatigue	342	2.72 (2.45-3.03)	2.67 (2.40-2.96)	359.83	1.41 (1.25)	2.66 (2.39)
Gastrointestinal disorders	Vomiting	225	3.56 (3.12-4.06)	3.50 (3.08-3.99)	404.27	1.79 (1.60)	3.50 (3.06)
Respiratory, thoracic and mediastinal disorders	Pneumonitis	188	42.42 (36.66-49.09)	41.65 (36.08-48.06)	7283.45	5.07 (4.86)	40.68 (35.15)
General disorders and administration site conditions	No adverse event	174	6.06 (5.22-7.04)	5.97 (5.15-6.92)	720.05	2.53 (2.31)	5.96 (5.13)
Metabolism and nutrition disorders	Decreased appetite	168	4.62 (3.97-5.39)	4.56 (3.93-5.30)	468.10	2.16 (1.93)	4.55 (3.91)
Skin and subcutaneous tissue disorders	Alopecia	153	4.96 (4.23-5.82)	4.90 (4.19-5.74)	475.58	2.25 (2.02)	4.89 (4.17)
Blood and lymphatic system disorders	Anemia	119	4.47 (3.73-5.35)	4.43 (3.70-5.29)	315.51	2.10 (1.84)	4.42 (3.69)
Blood and lymphatic system disorders	Neutropenia	117	4.59 (3.82-5.50)	4.54 (3.79-5.44)	323.34	2.14 (1.87)	4.53 (3.78)
Investigations	Neutrophil count decreased	102	14.06 (11.56-17.10)	13.93 (11.48-16.91)	1215.18	3.62 (3.33)	13.83 (11.37)
Blood and lymphatic system disorders	Thrombocytopenia	89	5.39 (4.37-6.64)	5.35 (4.35-6.58)	314.41	2.35 (2.04)	5.34 (4.33)
Blood and lymphatic system disorders	Febrile neutropenia	83	7.37 (5.94-9.16)	7.32 (5.91-9.08)	451.62	2.76 (2.45)	7.29 (5.87)
Investigations	Platelet count decreased	82	4.56 (3.67-5.67)	4.53 (3.65-5.62)	225.47	2.12 (1.80)	4.52 (3.64)
Neoplasms benign, malignant and unspecified (incl cysts and polyps)	Malignant neoplasm progression	75	4.18 (3.33-5.24)	4.15 (3.31-5.21)	179.48	1.99 (1.66)	4.15 (3.30)
Gastrointestinal disorders	Ascites	74	18.80 (14.94-23.66)	18.67 (14.86-23.45)	1224.33	3.91 (3.57)	18.47 (14.68)
Injury, poisoning and procedural complications	Underdose	72	6.68 (5.30-8.43)	6.64 (5.28-8.37)	344.25	2.62 (2.28)	6.62 (5.25)
Injury, poisoning and procedural complications	Toxicity to various agents	71	2.70 (2.13-3.40)	2.68 (2.13-3.38)	75.06	1.39 (1.05)	2.68 (2.12)
Respiratory, thoracic and mediastinal disorders	Pleural effusion	61	7.67 (5.96-9.87)	7.63 (5.94-9.80)	349.95	2.78 (2.41)	7.60 (5.90)
General disorders and administration site conditions	General physical health deterioration	58	3.11 (2.40-4.03)	3.10 (2.40-4.01)	82.52	1.58 (1.20)	3.10 (2.39)
Metabolism and nutrition disorders	Dehydration	51	2.99 (2.27-3.94)	2.98 (2.26-3.92)	67.03	1.52 (1.12)	2.98 (2.26)
Blood and lymphatic system disorders	Myelosuppression	49	6.49 (4.90-8.60)	6.46 (4.89-8.55)	225.62	2.54 (2.13)	6.44 (4.86)
Infections and infestations	Sepsis	47	2.93 (2.20-3.90)	2.92 (2.20-3.89)	59.37	1.49 (1.07)	2.92 (2.19)
Investigations	Ejection fraction decreased	47	16.70 (12.52-22.28)	16.63 (12.49-22.15)	684.01	3.64 (3.22)	16.48 (12.36)
Infections and infestations	Pneumocystis jirovecii pneumonia	39	20.62 (15.03-28.30)	20.55 (14.99-28.16)	716.73	3.78 (3.32)	20.31 (14.81)
Neoplasms benign, malignant and unspecified (incl cysts and polyps)	Metastases to central nervous system	37	17.78 (12.85-24.60)	17.72 (12.82-24.48)	577.85	3.61 (3.14)	17.55 (12.69)
Respiratory, thoracic and mediastinal disorders	Lung disorder	35	4.68 (3.36-6.53)	4.67 (3.35-6.51)	100.81	2.08 (1.60)	4.66 (3.34)

**Table 6:** The top signal strength of AEs of T-DXd ranked by EBGM at the PTs level.

SOC	PTs	Case reports	ROR (95% CI)	PRR (95% CI)	χ2	IC (IC025)	EBGM (EBGM05)
Infections and infestations	Gastroenteritis listeria	3	1023.85 (244.65-4284.84)	1023.54 (244.64-4282.42)	1915.40	1.99 (0.26)	640.09 (152.95)
Investigations	KL-6 increased	8	155.20 (75.24-320.15)	155.08 (75.22-319.73)	1122.66	3.09 (2.08)	142.24 (68.96)
Infections and infestations	Coronavirus pneumonia	3	77.56 (24.38-246.73)	77.54 (24.38-246.58)	216.81	1.94 (0.46)	74.21 (23.33)
Investigations	Cell marker increased	3	76.41 (24.03-242.96)	76.38 (24.03-242.81)	213.63	1.94 (0.46)	73.15 (23.01)
Neoplasms benign, malignant and unspecified (incl cysts and polyps)	Focal nodular hyperplasia	3	65.63 (20.71-207.97)	65.61 (20.71-207.84)	183.81	1.93 (0.45)	63.22 (19.95)
Hepatobiliary disorders	Pseudocirrhosis	3	63.99 (20.21-202.66)	63.97 (20.21-202.53)	179.24	1.93 (0.45)	61.70 (19.48)
Respiratory, thoracic and mediastinal disorders	Interstitial lung disease	430	62.12 (56.31-68.53)	59.51 (54.16-65.38)	23919.13	5.67 (5.52)	57.53 (52.15)
Surgical and medical procedures	Abdominal cavity drainage	4	58.85 (21.71-159.48)	58.82 (21.71-159.35)	219.79	2.22 (0.91)	56.90 (21.00)
Reproductive system and breast disorders	Breast hemorrhage	3	50.19 (15.92-158.23)	50.17 (15.92-158.14)	140.45	1.91 (0.44)	48.77 (15.47)
Neoplasms benign, malignant and unspecified (incl cysts and polyps)	Lymphangiosis carcinomatosa	7	45.09 (21.28-95.54)	45.06 (21.28-95.42)	293.83	2.79 (1.75)	43.93 (20.73)
Eye disorders	Eye hematoma	3	42.31 (13.45-133.04)	42.30 (13.45-132.96)	118.03	1.90 (0.43)	41.30 (13.13)
Respiratory, thoracic and mediastinal disorders	Pneumonitis	188	42.42 (36.66-49.09)	41.65 (36.08-48.06)	7283.45	5.07 (4.86)	40.68 (35.15)
Injury, poisoning and procedural complications	Radiation necrosis	4	40.39 (14.98-108.89)	40.38 (14.98-108.81)	150.05	2.18 (0.87)	39.47 (14.64)
Neoplasms benign, malignant and unspecified (incl cysts and polyps)	Metastases to bone marrow	3	39.38 (12.53-123.71)	39.37 (12.53-123.64)	109.65	1.89 (0.43)	38.50 (12.26)
Gastrointestinal disorders	Malignant ascites	3	36.57 (11.65-114.77)	36.56 (11.65-114.70)	101.57	1.88 (0.42)	35.81 (11.41)
Neoplasms benign, malignant and unspecified (incl cysts and polyps)	Metastases to skin	6	34.25 (15.26-76.87)	34.23 (15.26-76.79)	189.76	2.57 (1.47)	33.58 (14.96)
Metabolism and nutrition disorders	Fluid imbalance	3	30.84 (9.84-96.61)	30.83 (9.84-96.55)	85.05	1.86 (0.40)	30.30 (9.67)
Neoplasms benign, malignant and unspecified (incl cysts and polyps)	Metastases to meninges	12	30.68 (17.33-54.32)	30.65 (17.32-54.22)	338.07	3.22 (2.41)	30.12 (17.01)
Vascular disorders	Thrombophlebitis migrans	3	29.42 (9.40-92.13)	29.41 (9.40-92.07)	80.94	1.86 (0.40)	28.93 (9.24)
Respiratory, thoracic and mediastinal disorders	Lung opacity	22	28.38 (18.62-43.28)	28.32 (18.59-43.15)	570.49	3.68 (3.08)	27.88 (18.28)
Investigations	Tumor marker abnormal	3	27.52 (8.79-86.13)	27.51 (8.80-86.08)	75.44	1.85 (0.39)	27.09 (8.66)
Respiratory, thoracic and mediastinal disorders	Pulmonary toxicity	32	25.89 (18.25-36.73)	25.81 (18.21-36.57)	751.89	3.87 (3.36)	25.44 (17.93)
Infections and infestations	Pneumocystis jirovecii pneumonia	39	20.62 (15.03-28.30)	20.55 (14.99-28.16)	716.73	3.78 (3.32)	20.31 (14.81)
General disorders and administration site conditions	Disease progression	410	21.22 (19.21-23.43)	20.39 (18.54-22.43)	7487.07	4.27 (4.12)	20.16 (18.26)
Investigations	Eastern Cooperative Oncology Group performance status worsened	6	19.69 (8.81-44.05)	19.68 (8.80-44.01)	105.20	2.42 (1.32)	19.47 (8.70)
Gastrointestinal disorders	Ascites	74	18.80 (14.94-23.66)	18.67 (14.86-23.45)	1224.33	3.91 (3.57)	18.47 (14.68)
Infections and infestations	Hematological infection	6	17.87 (7.99-39.96)	17.86 (7.99-39.92)	94.53	2.39 (1.29)	17.69 (7.91)
Eye disorders	Keratitis	8	17.83 (8.88-35.79)	17.82 (8.88-35.75)	125.67	2.63 (1.66)	17.64 (8.79)
Neoplasms benign, malignant and unspecified (incl cysts and polyps)	Metastases to central nervous system	37	17.78 (12.85-24.60)	17.72 (12.82-24.48)	577.85	3.61 (3.14)	17.55 (12.69)
Metabolism and nutrition disorders	Cell death	6	17.66 (7.90-39.48)	17.65 (7.90-39.43)	93.26	2.38 (1.28)	17.48 (7.82)

## 4 Discussion

While aiming to extend survival, improving the quality of life remains a core goal in anticancer treatment. Minimizing the incidence and severity of AEs is crucial to ensuring patients can complete their treatment regimen (21, 22). Drug safety monitoring has always been a focus of post-marketing regulation. The FAERS database provides valuable information on drug safety, aiding in the dynamic monitoring of AEs. HER-2, as a target for treating the most prevalent BC globally, has been extensively validated. Additionally, HER-2 has also been targeted in the treatment of other common cancers like gastric, lung, and colorectal cancer, achieving significant therapeutic success. Therefore, in-depth analysis of the safety of anti-HER-2 drugs is vital, as revealing potentially overlooked AEs has potential reference value for the selection of clinical treatment plans. This study conducted an in-depth safety analysis of T-DXd, revealing its safety profile across a broad patient population based on FAERS data. The following discussion will delve deeper into the study’s results and findings.

### 4.1 Epidemiological characteristics of AEs

The study analyzed AE reports related to T-DXd from the FAERS database from the first quarter of 2020 to the third quarter of 2023. During this period, a total of 6,703,410 reports were recorded, with 4,055 related to T-DXd. The substantial volume of data provided a reliable sample for evaluating the safety profile of T-DXd and monitoring potential drug-related risks.

The number of AE reports from female patients was significantly higher than that from males, aligning with the gender distribution in breast cancer patients. This finding reiterates the epidemiological characteristic of breast cancer as a major malignant tumor in women and supports the necessity of focused drug safety and efficacy monitoring in female patients. A large proportion of reports had missing age data (61.09%), which might limit the comprehensive understanding of age-related AE risks. However, among the reports with provided age information, patients aged 45 to 65 years constituted the highest proportion, reflecting the trend of increasing breast cancer incidence with age (1). The annual increase in the number of reports could be associated with the rising use of T-DXd and heightened awareness of drug safety monitoring. Physicians were the primary reporters (58.91%), highlighting the critical role of healthcare professionals in drug safety monitoring. The geographical distribution of reports was predominantly from the United States (53.42%), possibly related to the FDA’s regulatory role, awareness of the AE reporting system, and higher drug usage rates. In this study, a relatively high proportion of serious AEs were noted, with 23.33% of reports involving death. This finding underlines the need for close monitoring of patients treated with T-DXd, especially during the initial phase of treatment. Furthermore, the first 30 days after medication was identified as a peak period for AEs, accounting for 13.51%, which is significant information for clinicians in monitoring patients at the onset of drug therapy.

### 4.2 Known AEs

In this study, some AEs associated with T-DXd were consistent with those recorded in the drug’s label, including General disorders and administration site conditions, Gastrointestinal disorders, and Respiratory, thoracic, and mediastinal disorders. These findings not only validate the information in the drug label but also emphasize the importance of monitoring these known AEs in clinical practice. High-frequency reports of AEs such as Nausea, Vomiting, Interstitial lung disease, Decreased appetite, and Alopecia suggest a need for clinical attention and the implementation of appropriate prevention and management measures. The AEs listed in the drug label are primarily based on clinical study results, and due to limitations in patient types and numbers, some delayed or rare AEs may not be identified promptly. Post-marketing studies based on spontaneous reporting data can provide more information for drug safety research.

### 4.3 Newly identified AE signals and their potential mechanisms

#### 4.3.1 Immune dysregulation

The strong signals of Gastroenteritis listeria and Coronavirus pneumonia might be related to the immunomodulatory effects of the drug. T-DXd could potentially reduce the patient’s defense against pathogens and disrupt gut microbiota balance (23), leading to Gastroenteritis listeria and Coronavirus pneumonia.

#### 4.3.2 Hepatobiliary disease-related AEs

AEs related to hepatobiliary systems identified in this study include Pseudocirrhosis, Hepatic failure, Ascites, and Malignant ascites.

##### 4.3.2.1 Pseudocirrhosis

In breast cancer patients, rapid tumor response and the shrinkage of liver metastases may lead to morphological changes in the liver. T-DXd might induce metabolic stress in the liver, causing structural alterations and potentially leading to fibrosis and architectural remodeling (24).

##### 4.3.2.2 Hepatic failure

The cytotoxic component in T-DXd may directly cause toxicity to liver cells, leading to cellular damage and functional failure (25). Additionally, the drug could trigger abnormal immune responses attacking liver tissue, resulting in inflammation and injury.

##### 4.3.2.3 Ascites and malignant ascites

Impaired liver function can lead to increased portal venous pressure, causing ascites. In the case of malignant ascites, direct invasion by the tumor or biologically active substances produced by the tumor may lead to fluid accumulation in the abdominal cavity. Furthermore, liver failure may reduce protein synthesis, causing a decrease in plasma colloidal osmotic pressure and prompting fluid leakage into the abdominal cavity.

#### 4.3.3 Vascular and hematological AEs

Neutropenia and Thrombocytopenia, associated with T-DXd treatment, may involve mechanisms where the drug induces abnormal activation of the immune system, leading to immune-mediated destruction of neutrophils (26). The cytotoxic components of T-DXd could also affect hematopoietic stem cells, impacting the production of neutrophils.

#### 4.3.4 Cancer progression-related AEs

Events such as Lymphangiosis carcinomatosa, Metastases to bone marrow, Metastases to skin, and Metastases to meninges are primarily related to the spread and metastasis of tumor cells. The mechanisms may involve the migration of tumor cells in the bloodstream, microenvironmental factors, and the breach of the blood-brain barrier (27). For patients receiving targeted therapies like T-DXd, the progression and metastasis of cancer remain significant concerns. Therefore, comprehensive patient assessment and monitoring of tumor metastasis risk are critical in clinical practice.

#### 4.3.5 Cardiovascular AEs

##### 4.3.5.1 Ejection fraction decreased

The components of T-DXd, especially the chemotherapeutic agents it carries, may exert direct toxic effects on the heart. These components can cause damage to myocardial cells, reducing the heart’s contractile function, leading to decreased ejection fraction (28). Additionally, the drug can cause vasodilation or other hemodynamic changes, indirectly affecting the heart’s pumping ability. Chemotherapeutic agents may also induce oxidative stress and inflammatory responses in cardiac tissue, further damaging myocardial cells.

##### 4.3.5.2 Cardiac dysfunction

Treatment with T-DXd may increase the cardiac workload, particularly in patients with a history of heart disease, which can lead to deterioration of cardiac function. The drug may cause coronary artery spasm or narrowing, leading to myocardial ischemia. Long-term use of T-DXd may gradually lead to a decline in cardiac function in some patients, especially in older individuals or those with cardiovascular disease risk factors (29).

#### 4.3.6 Ocular AEs

Ocular AEs related to T-DXd, such as Eye hematoma and Keratitis, may result from the direct toxic effects of the drug, immune-mediated inflammatory responses, or other physiological changes related to the drug therapy, such as coagulation dysfunction or blood pressure fluctuations.

### 4.4 Limitations

This study conducted an in-depth analysis of T-DXd-related AEs based on the FAERS database, which provided valuable real-world safety data across a large heterogeneous patient population, but several important limitations must be considered when interpreting the findings. First, as a spontaneous reporting system, FAERS is inherently subject to reporting biases (underreporting of mild events and overreporting of severe/novel events) and lacks formal control groups, preventing definitive causal inference. Second, critical confounding factors cannot be fully adjusted for: patients receiving T-DXd typically undergo multiple lines of therapy and use concomitant medications that may cause overlapping AEs; some high-signal events (e.g., malignant ascites, lymphangiosis carcinomatosa) are direct manifestations of advanced cancer progression rather than drug toxicities; and detailed information on baseline comorbidities, prior treatment history, and individual patient differences (genetic background, lifestyle) is not systematically recorded in FAERS. Additionally, our classification of novel AEs was based to data FDA prescribing information, and some signals may have been added to subsequent label updates. Although we used four complementary signal detection methods and required a minimum of 3 reported cases to reduce false-positive results, prospective cohort studies with detailed patient-level data are still needed to confirm the causal relationship between T-DXd and the identified novel AEs.

## 5 Conclusion

Through comprehensive analysis of data from the FAERS database, we identified a range of newly discovered AEs associated with T-DXd. These AEs span multiple domains, including immune dysregulation abnormalities, hepatobiliary system diseases, vascular and hematological issues, cardiovascular AEs, and ocular problems. These findings provide valuable safety information for clinicians using T-DXd to treat HER2-positive cancers. This information helps physicians better weigh the pros and cons in treatment decision-making, conduct targeted monitoring and management of patients to reduce the occurrence of AEs, optimize treatment outcomes, and improve the quality of life for patients. Additionally, this study offers direction for future drug safety research and clinical applications, emphasizing the importance of personalized medicine and long-term safety monitoring in modern cancer treatment.

## Data Availability

The raw data supporting the conclusions of this article will be made available by the authors, without undue reservation.
